# 1-(1*H*-Benzimidazol-1-ylmeth­yl)-3-[2-(di­isopropyl­amino)eth­yl]-1*H*-benzimid­azolium bromide 0.25-hydrate

**DOI:** 10.1107/S160053680900765X

**Published:** 2009-03-06

**Authors:** Hakan Arslan, Don VanDerveer, Serpil Demir, İsmail Özdemir, Bekir Çetinkaya

**Affiliations:** a Department of Natural Sciences, Fayetteville State University, Fayetteville, NC 28301, USA; b Department of Chemistry, Faculty of Pharmacy, Mersin University, Mersin TR 33169, Turkey; c Department of Chemistry, Clemson University, Clemson, SC 29634, USA; d Department of Chemistry, Faculty of Science and Arts, nönü University, Malatya TR 44280, Turkey; e Department of Chemistry, Faculty of Science, Ege University, Bornova-zmir TR 35100, Turkey

## Abstract

The title *N*-heterocyclic carbene derivative, C_23_H_30_N_5_^+^·Br^−^·0.25H_2_O, was synthesized using microwave heating and was characterized by ^1^H and ^13^C NMR spectroscopy and a single-crystal X-ray diffraction study. The structure of the title compound are stabilized by a network of intra- and inter­molecular C—H⋯Br hydrogen-bonding inter­actions. The crystal structure is further stabilized by π–π stacking inter­actions between benzene and imidazole fragment rings of parallel benzo[*d*]imidazole rings, with a separation of 3.486 (3) Å between the centroids of the benzene and imidazole rings. There is also an inter­molecular C—H⋯π inter­action in the crystal structure. The C—N bond lengths for the central benzimidazole ring are shorter than the average single C—N bond, thus showing varying degrees of double-bond character and indicating partial electron delocalization within the C—N—C—N—C fragment. The isopropyl group is disordered over two sites with occupancies of 0.792 (10) and 0.208 (10).

## Related literature

For the synthesis, see: Yaşar *et al.* (2008 ▶). For general background, see: Herrmann *et al.* (1995 ▶); Navarro *et al.* (2006 ▶); Arduengo & Krafczyc (1998 ▶); Larhed *et al.* (2002 ▶); Leadbeater & Shoemaker (2008 ▶). For related compounds, see: Özel Güven *et al.* (2008*a* ▶,*b* ▶,*c* ▶); Türktekin *et al.* (2004 ▶); Akkurt *et al.* (2004 ▶, 2005 ▶, 2007*a* ▶,*b* ▶); Arslan *et al.* (2005 ▶, 2007 ▶, 2009 ▶ and references therein).
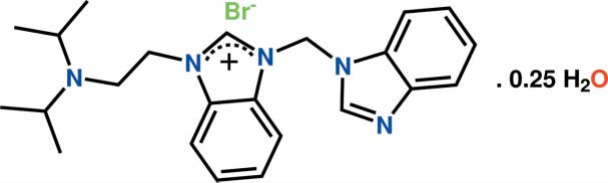


## Experimental

### 

#### Crystal data

C_23_H_30_N_5_^+^·Br^−^·0.25H_2_O
*M* *_r_* = 460.93
Triclinic, 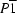

*a* = 8.4944 (17) Å
*b* = 9.4960 (19) Å
*c* = 15.318 (3) Å
α = 83.29 (3)°
β = 84.69 (3)°
γ = 65.93 (3)°
*V* = 1119.1 (5) Å^3^
*Z* = 2
Mo *K*α radiation
μ = 1.86 mm^−1^
*T* = 153 K
0.34 × 0.12 × 0.10 mm

#### Data collection

Rigaku Mercury CCD diffractometer
Absorption correction: multi-scan (*REQAB*; Jacobson, 1998 ▶) *T*_min_ = 0.571, *T*_max_ = 0.836
7638 measured reflections
3889 independent reflections
2390 reflections with *I* > 2σ(*I*)
*R*_int_ = 0.039

#### Refinement

*R*[*F* ^2^ > 2σ(*F* ^2^)] = 0.054
*wR*(*F* ^2^) = 0.144
*S* = 0.98
3889 reflections
282 parameters
22 restraints
H-atom parameters constrained
Δρ_max_ = 0.74 e Å^−3^
Δρ_min_ = −0.71 e Å^−3^

### 

Data collection: *CrystalClear* (Rigaku/MSC, 2006 ▶); cell refinement: *CrystalClear*; data reduction: *CrystalClear*; program(s) used to solve structure: *SHELXTL* (Sheldrick, 2008 ▶); program(s) used to refine structure: *SHELXTL*; molecular graphics: *SHELXTL*; software used to prepare material for publication: *SHELXTL*.

## Supplementary Material

Crystal structure: contains datablocks I, global. DOI: 10.1107/S160053680900765X/hg2484sup1.cif

Structure factors: contains datablocks I. DOI: 10.1107/S160053680900765X/hg2484Isup2.hkl

Additional supplementary materials:  crystallographic information; 3D view; checkCIF report

## Figures and Tables

**Table 1:** Hydrogen-bond geometry (Å, °)

*D*—H⋯*A*	*D*—H	H⋯*A*	*D*⋯*A*	*D*—H⋯*A*
C1—H1⋯Br1	0.96	2.75	3.493 (7)	135
C6—H6⋯Br1^i^	0.96	2.75	3.702 (6)	173
C20—H20*A*⋯*Cg*1	0.96	2.95	3.445 (5)	113

Symmetry code: (i) 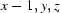. *Cg*1is the centroid of the N1,C1,N2,C7,C2 ring.

**Fig. 1.**
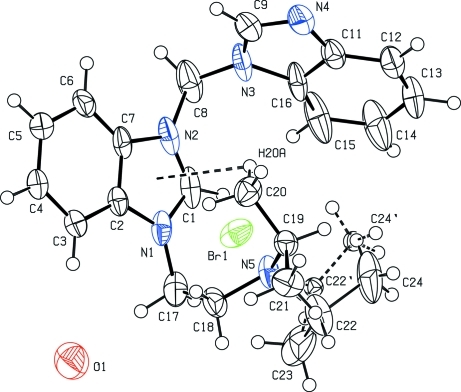
The molecular structure of the title compound, showing the atom-numbering scheme and displacement ellipsoids drawn at the 50% probability level.

**Fig. 2.**
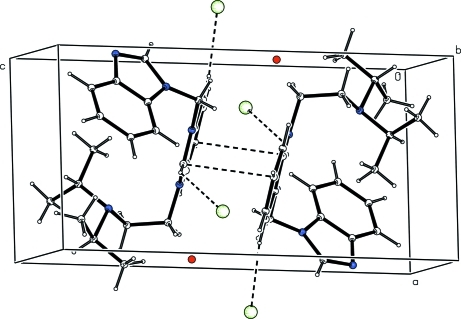
A packing diagram for (I).

**Fig. 3.**
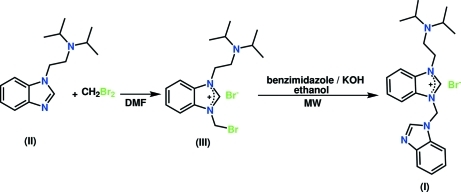
Synthesis of the title compound.

## supplementary crystallographic information

### Comment

N-Heterocyclic carbene compounds have been shown to have wide applicability in organometallic chemistry and catalysis such as Suzuki-Miyura, Sonogashira, Stille and Heck reactions (Herrmann *et al.*, 1995; Navarro *et al.*, 2006; Arduengo & Krafczyc, 1998). In general, N-heterocyclic carbene chemistry is dominated by imidazole, diazepin, benzimidazole and their derivatives based carbene ligands.

Microwave-promoted synthesis is an area of increasing interest in both academic and industrial laboratories (Larhed *et al.*, 2002). Microwave heating offers a fast, easy way to perform chemical reactions that require heat. Synthetic organic chemists have taken advantage of microwave heating in their work and found that reaction times can often be reduced from hours to minutes with a significant improvement in yields (Leadbeater & Shoemaker, 2008).

Our team has been interested in complexes of derivatives based on N-heterocyclic carbene compounds which exhibit high catalytic activities for Suzuki-Miyura, and Heck reactions. As a continuation of our systematic studies of the various N-heterocyclic carbene compounds and the catalytic properties of their palladium, ruthenium and rhodium complexes (Yaşar *et al.*, 2008; Arslan *et al.*, 2005, 2007, 2009, and references therein), we have prepared a new carbene compound which includes a benzo[*d*]imidazole and an amine group. The title compound, (I), synthesis and characterization, including its crystal structure is reported here. The compound was purified by re-crystallizationfrom an ethanol:diethylether mixture (1:2) and characterized by ^1^H and ^13^C-NMR. These data are consistent with the proposed structure given in Scheme 1.

The crystallographic asymmetric unit of the title compound contains a single 3-((1*H*-benzo[*d*]imidazol-1-yl)methyl)-1-(2-(diisopropylamino)ethyl)-1*H*-benzo[*d*]imidazol-3-ium cation, one bromide anion and 0.25 mole water molecule linked by hydrogen and stacking interactions to form a three-dimensional framework. The molecular structure of the title compound is depicted in Fig. 1.

The imidazole and benzimidazole ring systems are essentially planar with maximum deviations of 0.002 (5), 0.008 (5), 0.029 (5) and 0.020 (5) Å for N1—C1—N2—C7—C2, N3—C9—N4—C11—C16, N1—C1—N2—C7—C2—C3—C4—C5—C6 and N3—C9—N4—C11—C12—C13—C14—C15 rings, respectively. The dihedral angle between the benzimidazole rings is 69.51 (8)*^o^*. The geometric parameters for the N3—C9—N4—C11—C12—C13—C14—C15 benzimidazole ring agree with the other reported benzimidazole derivatives (Özel Güven *et al.*, 2008*a*, 2008*b*, 2008*c*; Türktekin *et al.*, 2004; Akkurt *et al.*, 2004, 2005, 2007*a*, 2007*b*). In particular, in the N—C—N fragments, the C9—N4 bond length (1.295 (8) Å) is *ca* 0.08 Å shorter than the C9—N3 bond length (1.367 (9) Å), which is consistent with the partial double-bond character. The C—N bond lengths for the other benzimidazole ring are shorter than the average single C—N bond, being N1—C1 = 1.332 (7) Å, N2—C1 = 1.332 (9) Å, N1—C2 = 1.398 (7) Å, and N2—C7 = 1.399 (6) Å thus showing varying degrees of double bond character in these C—N bonds. This information indicates a partial electron delocalization within the C2—N1—C1—N2—C7 fragment. This result is confirmed by the N1—C1—N2 bond angle.

The crystal packing is stabilized mainly by C—H···Br hydrogen bonds and stacking interactions. A partially overlapped arrangement is observed between parallel benzimidazole rings (see Fig. 2) so these parallel benzimidazole rings are linked by π-π stacking interactions. The centroid-centroid separation between the parallel imidazole and benzene ring fragments (N1—C1—N2—C7—C2*^i ^*and C2—C3—C4—C5—C6—C7*^i^*) of the benzimidazole ring is 3.486 (3) Å with C1···C4*^ii^* = 3.398 (7) Å [symmetry code: (*i*) *x*, *y*, *z*, (*ii*) 1 - *x*, 1 - *y*, 1 - *z*]. In addition, a C—H···π interaction is observed between *Cg*1 (Centroid of N1—C1—N2—C7—C2 ring) and the C20 atom: H20A···*Cg*1*^i^* = 2.950 Å, C20—H20A···*Cg*1*^i^* = 113.0° [symmetry code: (*i*) *x*, *y*, *z*].

### Experimental

All reactions for the preparation of (II) and (III) were carried out under Ar inflame-dried glass-ware using standard Schlenk-type flasks (Fig. 3). All ^1^H and ^13^C-NMRs were performed in CDCl_3. _^1^H NMR and ^13^C NMR spectra were recorded using a Varian As 400 Merkur spectrometer operating at 400 MHz (^1^H) and 100 MHz (^13^C). Chemical shifts (δ) are given in p.p.m. relative to TMS, coupling constants (*J*) in Hz. Melting points were measured in open capillary tubes with an Electrothermal-9200 melting point apparatus and are uncorrected. Microwave assisted reactions were carried out in a self-tuning single mode CEM Discover microwave unit. This consist of a continuous focused microwave power delivery system with operator-selectable power output from 0 to 300 W. The reaction was performed in an 80 ml capacity sealed tube. Temperature, pressure and power profiles were monitored using commercially available software provided by the microwave manufacturer.

Dibromomethane (1.74 g, 10.0 mmol) was slowly added to a solution of *N*-(2-(1*H*-benzo[*d*]imidazol-1-yl)ethyl)-*N*-isopropylpropan-2-amine (II) (2.45 g, 10.0 mmol) in DMF (5 ml) and the resulting mixture was stirred at 50 *^o^*C for 5 h (Fig. 3). Diethylether (10 ml) was added to obtain a white crystalline solid which was filtered off. The solid was washed with diethylether (3x10 ml), dried under vacuum and the crude product (III) was recrystallized from ethanol:diethylether. The yield was 2.72 g, 65%. In a dry 80 ml glass vessel equipped with a magnetic stirbar were added a potassium hydroxide (1 mmol) solution of benzimidazole (1 mmol) in ethanol (20 ml) and compound (III) (1 mmol). The vessel was sealed with a septum and placed in the microwave apparatus. With stirring, the reaction mixture was heated to 100 *^o^*C using an initial microwave power of 300 W and was held at this temperature for 10 min. The reaction mixture was then cooled to 50 *^o^*C, the solid was filtered off. The solvent was removed under vacuum. The product (I) was recrystallized from ethanol:diethylether (1: 2 ratio). The yield was 3.47 g, 76%, *M*.p.= 208–209 *^o^*C. ^1^H NMR (δ, 399.9 MHz, CDCl_3_): 0.87 (d, 12H, *J=* 6.6 Hz, NCH(C*H*_3_)_2_), 2.98 (t, 2H, *J =* 6.0 Hz, NCH_2_C*H*_2_N), 3.04 (hept, 2H, *J =* 6.6 Hz, NC*H*(CH_3_)_2_), 4.58 (t, 2H, *J =* 6.0 Hz, NCH_2_C*H*_2_N), 5.98 (s, 2H, –C*H*_2_–), 7.61–7.77 (m, 9H, C_6_*H*_4 _and NC*H*=N), 10.96 (s, 1H, NC*H*N). ^13^C NMR (δ, CDCl_3_): 20.8 (NCH(*C*H_3_)_2_), 44.5 (N*C*H_2_*C*H_2_N), 47.8 (N*C*H(CH_3_)_2_), 48.2 (-*C*H_2_–), 112.7, 113.2, 126.4, 126.7, 130.5 and 131.4 (*C*_6_H_4_), 143.4 (N*C*H=N), 143.6 (N*C*HN).

### Refinement

The H atoms were geometrically placed and treated as riding atoms with C—H = 0.96 Å, and *U*_iso_(H) = 1.5 *U*_eq_ (parent C-atom = CH_3_). The other H atoms were treated the same with *U*_iso_(H) = 1.2 *U*_eq_ (parent C-atom). We were unable to assign H atoms to the water molecule.

The isopropyl group (C22, C23, C24) is disordered. We were able to resolve C22 and C24 into two atoms. The major/minor component ratio is 0.79/0.21. The two minor component atoms were refined isotropically.

### Crystal data

**Table:**

C_23_H_30_N_5_^+^·Br^−^·0.25H_2_O	*Z* = 2
*M**_r_* = 460.93	*F*(000) = 481
Triclinic, *P*1	*D*_x_ = 1.368 Mg m^−^^3^
Hall symbol: -P 1	Mo *K*α radiation, λ = 0.71073 Å
*a* = 8.4944 (17) Å	Cell parameters from 5869 reflections
*b* = 9.4960 (19) Å	θ = 3.2–26.4°
*c* = 15.318 (3) Å	µ = 1.86 mm^−^^1^
α = 83.29 (3)°	*T* = 153 K
β = 84.69 (3)°	Rod, colorless
γ = 65.93 (3)°	0.34 × 0.12 × 0.10 mm
*V* = 1119.1 (5) Å^3^	

### Data collection

**Table:**

Rigaku Mercury CCD diffractometer	3889 independent reflections
Radiation source: Sealed Tube	2390 reflections with *I* > 2σ(*I*)
Graphite Monochromator	*R*_int_ = 0.039
Detector resolution: 14.6306 pixels mm^-1^	θ_max_ = 25.0°, θ_min_ = 3.2°
ω scans	*h* = −9→10
Absorption correction: multi-scan (REQAB; Jacobson, 1998)	*k* = −11→11
*T*_min_ = 0.571, *T*_max_ = 0.836	*l* = −17→18
7638 measured reflections	

### Refinement

**Table:**

Refinement on *F*^2^	Primary atom site location: structure-invariant direct methods
Least-squares matrix: full	Secondary atom site location: difference Fourier map
*R*[*F*^2^ > 2σ(*F*^2^)] = 0.054	Hydrogen site location: inferred from neighbouring sites
*wR*(*F*^2^) = 0.144	H-atom parameters constrained
*S* = 0.98	*w* = 1/[σ^2^(*F*_o_^2^) + (0.0659*P*)^2^] where *P* = (*F*_o_^2^ + 2*F*_c_^2^)/3
3889 reflections	(Δ/σ)_max_ = 0.001
282 parameters	Δρ_max_ = 0.74 e Å^−^^3^
22 restraints	Δρ_min_ = −0.71 e Å^−^^3^

### Special details

**Table:**

Geometry. All e.s.d.'s (except the e.s.d. in the dihedral angle between two l.s. planes) are estimated using the full covariance matrix. The cell e.s.d.'s are taken into account individually in the estimation of e.s.d.'s in distances, angles and torsion angles; correlations between e.s.d.'s in cell parameters are only used when they are defined by crystal symmetry. An approximate (isotropic) treatment of cell e.s.d.'s is used for estimating e.s.d.'s involving l.s. planes.
Refinement. Refinement of *F*^2^ against ALL reflections. The weighted *R*-factor *wR* and goodness of fit *S* are based on *F*^2^, conventional *R*-factors *R* are based on *F*, with *F* set to zero for negative *F*^2^. The threshold expression of *F*^2^ > σ(*F*^2^) is used only for calculating *R*-factors(gt) *etc*. and is not relevant to the choice of reflections for refinement. *R*-factors based on *F*^2^ are statistically about twice as large as those based on *F*, and *R*- factors based on ALL data will be even larger.

### Fractional atomic coordinates and isotropic or equivalent isotropic displacement parameters (Å^2^)

**Table:**

	*x*	*y*	*z*	*U*_iso_*/*U*_eq_	Occ. (<1)
Br1	0.78369 (7)	0.88546 (6)	0.57962 (4)	0.05288 (19)	
N1	0.6315 (5)	0.5169 (5)	0.6422 (2)	0.0430 (10)	
N2	0.3772 (6)	0.7103 (4)	0.6314 (3)	0.0499 (11)	
N3	0.1831 (7)	0.9149 (5)	0.7189 (3)	0.0544 (12)	
N4	0.0314 (6)	0.9404 (5)	0.8478 (3)	0.0575 (12)	
N5	0.7558 (5)	0.4797 (5)	0.8187 (2)	0.0411 (10)	
C1	0.5416 (8)	0.6676 (6)	0.6495 (3)	0.0511 (14)	
H1	0.5890	0.7364	0.6658	0.061*	
C2	0.5188 (6)	0.4562 (5)	0.6178 (3)	0.0334 (10)	
C3	0.5464 (6)	0.3083 (5)	0.5999 (3)	0.0352 (10)	
H3	0.6568	0.2231	0.6057	0.042*	
C4	0.4050 (6)	0.2909 (5)	0.5732 (3)	0.0375 (11)	
H4	0.4179	0.1906	0.5595	0.045*	
C5	0.2451 (6)	0.4136 (5)	0.5654 (3)	0.0416 (12)	
H5	0.1508	0.3949	0.5470	0.050*	
C6	0.2162 (7)	0.5628 (6)	0.5833 (3)	0.0475 (13)	
H6	0.1060	0.6480	0.5770	0.057*	
C7	0.3565 (7)	0.5795 (5)	0.6106 (3)	0.0382 (11)	
C8	0.2416 (10)	0.8685 (6)	0.6319 (4)	0.0700 (18)	
H8A	0.2855	0.9397	0.6007	0.084*	
H8B	0.1452	0.8743	0.6013	0.084*	
C9	0.0487 (8)	0.8983 (6)	0.7688 (4)	0.0629 (17)	
H9	−0.0264	0.8588	0.7471	0.075*	
C11	0.1667 (7)	0.9865 (5)	0.8532 (3)	0.0416 (12)	
C12	0.2084 (7)	1.0432 (5)	0.9234 (3)	0.0470 (13)	
H12	0.1438	1.0529	0.9788	0.056*	
C13	0.3479 (9)	1.0850 (7)	0.9095 (4)	0.0683 (17)	
H13	0.3776	1.1281	0.9559	0.082*	
C14	0.4463 (10)	1.0666 (8)	0.8304 (5)	0.091 (2)	
H14	0.5443	1.0937	0.8245	0.109*	
C15	0.4059 (9)	1.0098 (7)	0.7597 (4)	0.078 (2)	
H15	0.4722	0.9984	0.7047	0.093*	
C16	0.2652 (8)	0.9710 (5)	0.7730 (3)	0.0497 (14)	
C17	0.8119 (6)	0.4275 (7)	0.6628 (3)	0.0518 (14)	
H17A	0.8739	0.4932	0.6528	0.062*	
H17B	0.8634	0.3453	0.6247	0.062*	
C18	0.8254 (7)	0.3596 (6)	0.7583 (3)	0.0461 (13)	
H18A	0.7636	0.2936	0.7680	0.055*	
H18B	0.9444	0.2974	0.7696	0.055*	
C19	0.6381 (6)	0.4499 (5)	0.8896 (3)	0.0348 (10)	
H19	0.6113	0.5273	0.9302	0.042*	
C20	0.4689 (6)	0.4737 (6)	0.8519 (3)	0.0462 (12)	
H20A	0.4186	0.5761	0.8229	0.069*	
H20B	0.3911	0.4610	0.8986	0.069*	
H20C	0.4903	0.3989	0.8103	0.069*	
C21	0.7152 (7)	0.2931 (6)	0.9424 (3)	0.0489 (13)	
H21A	0.7476	0.2122	0.9037	0.073*	
H21B	0.6313	0.2831	0.9864	0.073*	
H21C	0.8153	0.2852	0.9705	0.073*	
C22	0.9092 (11)	0.5022 (9)	0.8612 (5)	0.054 (2)	0.792 (10)
H22	0.9695	0.4146	0.9013	0.065*	0.792 (10)
C22'	0.843 (2)	0.582 (2)	0.8331 (14)	0.021 (5)*	0.208 (10)
H22'	0.9455	0.5571	0.8048	0.025*	0.208 (10)
C23	1.0298 (9)	0.5239 (10)	0.7878 (5)	0.098 (2)	
H23A	1.1470	0.4660	0.8038	0.118*	
H23B	1.0083	0.6318	0.7774	0.118*	
H23C	1.0110	0.4880	0.7353	0.118*	
C24	0.8241 (13)	0.6470 (11)	0.9089 (6)	0.090 (3)	0.792 (10)
H24A	0.7210	0.7156	0.8803	0.136*	0.792 (10)
H24B	0.9019	0.6974	0.9079	0.136*	0.792 (10)
H24C	0.7950	0.6202	0.9687	0.136*	0.792 (10)
C24'	0.729 (2)	0.726 (2)	0.8681 (14)	0.022 (5)*	0.208 (10)
H24D	0.6329	0.7782	0.8312	0.034*	0.208 (10)
H24E	0.7908	0.7908	0.8697	0.034*	0.208 (10)
H24F	0.6878	0.7052	0.9265	0.034*	0.208 (10)
O1	0.968 (2)	0.133 (2)	0.5686 (12)	0.076 (5)	0.25

### Atomic displacement parameters (Å^2^)

**Table:**

	*U*^11^	*U*^22^	*U*^33^	*U*^12^	*U*^13^	*U*^23^
Br1	0.0484 (3)	0.0364 (3)	0.0800 (4)	−0.0213 (2)	−0.0238 (3)	0.0042 (2)
N1	0.061 (2)	0.061 (3)	0.024 (2)	−0.042 (2)	0.0074 (18)	−0.0127 (18)
N2	0.093 (3)	0.030 (2)	0.028 (2)	−0.025 (2)	−0.002 (2)	−0.0085 (18)
N3	0.097 (4)	0.031 (2)	0.030 (2)	−0.017 (2)	−0.011 (2)	−0.0116 (18)
N4	0.054 (3)	0.050 (3)	0.068 (3)	−0.015 (2)	−0.003 (2)	−0.027 (2)
N5	0.058 (2)	0.051 (2)	0.026 (2)	−0.033 (2)	0.0081 (18)	−0.0133 (18)
C1	0.097 (4)	0.051 (3)	0.026 (3)	−0.050 (3)	0.006 (3)	−0.011 (2)
C2	0.045 (2)	0.038 (3)	0.025 (2)	−0.025 (2)	0.007 (2)	−0.0089 (19)
C3	0.042 (2)	0.035 (2)	0.030 (3)	−0.017 (2)	0.004 (2)	−0.008 (2)
C4	0.051 (3)	0.036 (3)	0.032 (3)	−0.023 (2)	0.000 (2)	−0.009 (2)
C5	0.041 (3)	0.044 (3)	0.041 (3)	−0.016 (2)	−0.002 (2)	−0.012 (2)
C6	0.053 (3)	0.046 (3)	0.028 (3)	−0.002 (3)	−0.003 (2)	−0.014 (2)
C7	0.064 (3)	0.034 (3)	0.019 (2)	−0.020 (2)	−0.002 (2)	−0.0061 (19)
C8	0.128 (5)	0.032 (3)	0.038 (3)	−0.017 (3)	−0.014 (3)	−0.004 (2)
C9	0.057 (3)	0.048 (3)	0.077 (5)	−0.006 (3)	−0.009 (3)	−0.032 (3)
C11	0.058 (3)	0.027 (2)	0.036 (3)	−0.012 (2)	−0.003 (2)	−0.005 (2)
C12	0.068 (3)	0.034 (3)	0.035 (3)	−0.015 (3)	−0.005 (3)	−0.006 (2)
C13	0.100 (5)	0.065 (4)	0.055 (4)	−0.042 (4)	0.002 (3)	−0.033 (3)
C14	0.129 (5)	0.098 (5)	0.088 (5)	−0.088 (4)	0.046 (4)	−0.058 (4)
C15	0.145 (5)	0.067 (4)	0.053 (4)	−0.078 (4)	0.041 (4)	−0.031 (3)
C16	0.097 (4)	0.027 (3)	0.031 (3)	−0.029 (3)	−0.004 (3)	−0.009 (2)
C17	0.054 (3)	0.081 (4)	0.041 (3)	−0.047 (3)	0.014 (2)	−0.024 (3)
C18	0.048 (3)	0.057 (3)	0.036 (3)	−0.022 (3)	0.011 (2)	−0.019 (2)
C19	0.038 (2)	0.040 (3)	0.026 (2)	−0.014 (2)	0.0014 (19)	−0.004 (2)
C20	0.047 (3)	0.060 (3)	0.036 (3)	−0.025 (3)	−0.002 (2)	−0.002 (2)
C21	0.051 (3)	0.044 (3)	0.044 (3)	−0.014 (3)	0.000 (2)	0.006 (2)
C22	0.092 (5)	0.050 (4)	0.035 (4)	−0.047 (4)	0.021 (4)	−0.008 (3)
C23	0.076 (4)	0.142 (6)	0.109 (6)	−0.067 (5)	−0.014 (4)	−0.037 (5)
C24	0.131 (7)	0.119 (7)	0.075 (6)	−0.100 (6)	0.029 (6)	−0.053 (5)
O1	0.070 (10)	0.081 (11)	0.091 (13)	−0.040 (9)	0.006 (9)	−0.027 (10)

### Geometric parameters (Å, °)

**Table:**

N1—C1	1.332 (6)	C14—C15	1.392 (8)
N1—C2	1.399 (5)	C14—H14	0.9600
N1—C17	1.461 (7)	C15—C16	1.379 (8)
N2—C1	1.333 (7)	C15—H15	0.9600
N2—C7	1.398 (5)	C17—C18	1.522 (7)
N2—C8	1.474 (7)	C17—H17A	0.9600
N3—C9	1.367 (7)	C17—H17B	0.9600
N3—C16	1.404 (6)	C18—H18A	0.9600
N3—C8	1.432 (7)	C18—H18B	0.9600
N4—C9	1.296 (7)	C19—C20	1.519 (6)
N4—C11	1.399 (6)	C19—C21	1.524 (7)
N5—C18	1.450 (6)	C19—H19	0.9600
N5—C22'	1.487 (19)	C20—H20A	0.9599
N5—C19	1.489 (5)	C20—H20B	0.9599
N5—C22	1.608 (8)	C20—H20C	0.9599
C1—H1	0.9600	C21—H21A	0.9599
C2—C3	1.382 (6)	C21—H21B	0.9599
C2—C7	1.402 (7)	C21—H21C	0.9599
C3—C4	1.380 (6)	C22—C23	1.504 (9)
C3—H3	0.9600	C22—C24	1.507 (10)
C4—C5	1.387 (6)	C22—H22	0.9600
C4—H4	0.9600	C22—H22'	1.045 (6)
C5—C6	1.393 (6)	C22'—C24'	1.44 (2)
C5—H5	0.9600	C22'—H22'	0.889 (17)
C6—C7	1.372 (7)	C23—H22'	0.693 (7)
C6—H6	0.9600	C23—H23A	0.9600
C8—H8A	0.9600	C23—H23B	0.9600
C8—H8B	0.9600	C23—H23C	0.9600
C9—H9	0.9600	C24—H24A	0.9600
C11—C12	1.391 (7)	C24—H24B	0.9600
C11—C16	1.407 (7)	C24—H24C	0.9600
C12—C13	1.387 (8)	C24'—H24D	0.9600
C12—H12	0.9600	C24'—H24E	0.9600
C13—C14	1.392 (8)	C24'—H24F	0.9600
C13—H13	0.9600		
			
C1—N1—C2	107.8 (4)	C15—C16—N3	133.2 (5)
C1—N1—C17	126.4 (4)	C15—C16—C11	122.7 (5)
C2—N1—C17	125.6 (4)	N3—C16—C11	104.0 (5)
C1—N2—C7	108.1 (4)	N1—C17—C18	110.7 (4)
C1—N2—C8	125.8 (5)	N1—C17—H17A	109.5
C7—N2—C8	126.1 (5)	C18—C17—H17A	109.5
C9—N3—C16	106.5 (4)	N1—C17—H17B	109.5
C9—N3—C8	127.1 (5)	C18—C17—H17B	109.5
C16—N3—C8	126.1 (5)	H17A—C17—H17B	108.1
C9—N4—C11	104.7 (5)	N5—C18—C17	111.7 (4)
C18—N5—C22'	123.0 (8)	N5—C18—H18A	109.3
C18—N5—C19	113.1 (3)	C17—C18—H18A	109.3
C22'—N5—C19	119.4 (8)	N5—C18—H18B	109.3
C18—N5—C22	110.5 (4)	C17—C18—H18B	109.3
C19—N5—C22	109.9 (3)	H18A—C18—H18B	108.0
N1—C1—N2	111.1 (4)	N5—C19—C20	110.0 (4)
N1—C1—H1	124.5	N5—C19—C21	114.8 (4)
N2—C1—H1	124.5	C20—C19—C21	111.1 (4)
C3—C2—N1	131.3 (4)	N5—C19—H19	106.8
C3—C2—C7	121.9 (4)	C20—C19—H19	106.8
N1—C2—C7	106.8 (4)	C21—C19—H19	106.8
C4—C3—C2	115.7 (4)	C19—C20—H20A	109.5
C4—C3—H3	122.1	C19—C20—H20B	109.5
C2—C3—H3	122.1	H20A—C20—H20B	109.5
C3—C4—C5	122.2 (4)	C19—C20—H20C	109.5
C3—C4—H4	118.9	H20A—C20—H20C	109.5
C5—C4—H4	118.9	H20B—C20—H20C	109.5
C4—C5—C6	122.4 (4)	C19—C21—H21A	109.5
C4—C5—H5	118.8	C19—C21—H21B	109.5
C6—C5—H5	118.8	H21A—C21—H21B	109.5
C7—C6—C5	115.3 (5)	C19—C21—H21C	109.5
C7—C6—H6	122.4	H21A—C21—H21C	109.5
C5—C6—H6	122.4	H21B—C21—H21C	109.5
C6—C7—N2	131.2 (5)	C23—C22—C24	110.2 (6)
C6—C7—C2	122.5 (4)	C23—C22—N5	108.3 (5)
N2—C7—C2	106.3 (4)	C24—C22—N5	105.8 (6)
N3—C8—N2	113.0 (4)	C23—C22—H22	110.8
N3—C8—H8A	109.0	C24—C22—H22	110.8
N2—C8—H8A	109.0	N5—C22—H22	110.8
N3—C8—H8B	109.0	C24'—C22'—N5	114.1 (14)
N2—C8—H8B	109.0	N5—C22'—H22'	113.3 (17)
H8A—C8—H8B	107.8	C22—C23—H23A	109.5
N4—C9—N3	114.3 (5)	C22—C23—H23B	109.5
N4—C9—H9	122.9	H23A—C23—H23B	109.5
N3—C9—H9	122.9	C22—C23—H23C	109.5
C12—C11—N4	129.0 (5)	H23A—C23—H23C	109.5
C12—C11—C16	120.5 (5)	H23B—C23—H23C	109.5
N4—C11—C16	110.5 (4)	C22—C24—H24A	109.5
C13—C12—C11	116.8 (5)	C22—C24—H24B	109.5
C13—C12—H12	121.6	H24A—C24—H24B	109.5
C11—C12—H12	121.6	C22—C24—H24C	109.5
C12—C13—C14	122.2 (5)	H24A—C24—H24C	109.5
C12—C13—H13	118.9	H24B—C24—H24C	109.5
C14—C13—H13	118.9	C22'—C24'—H24D	109.5
C13—C14—C15	121.5 (6)	C22'—C24'—H24E	109.5
C13—C14—H14	119.3	H24D—C24'—H24E	109.5
C15—C14—H14	119.3	C22'—C24'—H24F	109.5
C16—C15—C14	116.3 (5)	H24D—C24'—H24F	109.5
C16—C15—H15	121.9	H24E—C24'—H24F	109.5
C14—C15—H15	121.9		
			
C2—N1—C1—N2	−0.1 (5)	C11—C12—C13—C14	−2.2 (9)
C17—N1—C1—N2	−175.5 (4)	C12—C13—C14—C15	2.3 (11)
C7—N2—C1—N1	0.0 (5)	C13—C14—C15—C16	−1.0 (10)
C8—N2—C1—N1	−179.2 (4)	C14—C15—C16—N3	178.3 (6)
C1—N1—C2—C3	178.5 (5)	C14—C15—C16—C11	−0.2 (9)
C17—N1—C2—C3	−6.1 (7)	C9—N3—C16—C15	−179.9 (6)
C1—N1—C2—C7	0.2 (5)	C8—N3—C16—C15	6.3 (9)
C17—N1—C2—C7	175.6 (4)	C9—N3—C16—C11	−1.1 (5)
N1—C2—C3—C4	−176.9 (4)	C8—N3—C16—C11	−174.9 (5)
C7—C2—C3—C4	1.1 (6)	C12—C11—C16—C15	0.2 (8)
C2—C3—C4—C5	−0.5 (7)	N4—C11—C16—C15	179.3 (5)
C3—C4—C5—C6	0.5 (7)	C12—C11—C16—N3	−178.7 (4)
C4—C5—C6—C7	−1.1 (7)	N4—C11—C16—N3	0.4 (5)
C5—C6—C7—N2	177.7 (5)	C1—N1—C17—C18	90.7 (5)
C5—C6—C7—C2	1.7 (7)	C2—N1—C17—C18	−83.9 (5)
C1—N2—C7—C6	−176.3 (5)	C22'—N5—C18—C17	−71.1 (11)
C8—N2—C7—C6	2.9 (8)	C19—N5—C18—C17	133.1 (4)
C1—N2—C7—C2	0.1 (5)	C22—N5—C18—C17	−103.2 (5)
C8—N2—C7—C2	179.4 (4)	N1—C17—C18—N5	−61.6 (5)
C3—C2—C7—C6	−1.8 (7)	C18—N5—C19—C20	−71.9 (5)
N1—C2—C7—C6	176.7 (4)	C22'—N5—C19—C20	131.3 (10)
C3—C2—C7—N2	−178.7 (4)	C22—N5—C19—C20	164.0 (4)
N1—C2—C7—N2	−0.2 (5)	C18—N5—C19—C21	54.3 (5)
C9—N3—C8—N2	−91.4 (7)	C22'—N5—C19—C21	−102.6 (10)
C16—N3—C8—N2	81.2 (7)	C22—N5—C19—C21	−69.8 (5)
C1—N2—C8—N3	−74.6 (7)	C18—N5—C22—C23	51.6 (7)
C7—N2—C8—N3	106.3 (6)	C22'—N5—C22—C23	−68.6 (15)
C11—N4—C9—N3	−1.3 (6)	C19—N5—C22—C23	177.2 (5)
C16—N3—C9—N4	1.6 (7)	C18—N5—C22—C24	169.8 (5)
C8—N3—C9—N4	175.3 (5)	C22'—N5—C22—C24	49.5 (15)
C9—N4—C11—C12	179.6 (5)	C19—N5—C22—C24	−64.6 (6)
C9—N4—C11—C16	0.5 (6)	C18—N5—C22'—C24'	158.1 (13)
N4—C11—C12—C13	−178.0 (5)	C19—N5—C22'—C24'	−47.5 (19)
C16—C11—C12—C13	1.0 (7)	C22—N5—C22'—C24'	−127 (3)

### Hydrogen-bond geometry (Å, °)

**Table:**

*D*—H···*A*	*D*—H	H···*A*	*D*···*A*	*D*—H···*A*
C1—H1···Br1	0.96	2.75	3.493 (7)	135
C6—H6···Br1^i^	0.96	2.75	3.702 (6)	173
C20—H20A···Cg1	0.96	2.95	3.445 (5)	113

Symmetry codes: (i) *x*−1, *y*, *z*.
